# Effects of different glide path techniques on the amount of extruded debris and preparation times during root canal preparation

**DOI:** 10.34172/joddd.2020.031

**Published:** 2020-09-21

**Authors:** Damla Kırıcı, Simay Koç, Alper Kuştarcı

**Affiliations:** ^1^Department of Endodontics, Faculty of Dentistry, Akdeniz University, Antalya, Turkey

**Keywords:** WaveOne Gold Glider, ProGlider, WaveOne Gold, Glide path, Preparation time

## Abstract

**Background.** This study purposed to compare the effect of new single glide path files on extruded apical debris and total preparation times during root canal preparation with the WaveOne Gold system.

**Methods.** Thirty-six extracted human lower molar teeth with mesiobuccal canal curvature angles of 25‒35° were randomly splited to three groups. In group 1, the glide path was created with WaveOne Gold Glider (WGG) file at working length (WL); in group 2, the glide path was created with ProGlider file (PG); in group 3, the glide path was not performed. In all the groups, the root canals were shaped with WaveOne Gold Primary (WOG) reciprocating files at WL. Apically extruded debris during instrumentation was picked up into pre-weighed Eppendorf tubes. The weight of the dry extruded debris was calculated by subtracting the pre- and post-instrumentation weights of the tubes in each group. The total time elapsed during the canal preparation was calculated with a chronometer. The data were analyzed using one-way ANOVA and post hoc Tukey tests.

**Results.** The WGG/WOG group extruded significantly fewer debris than the WOG and PG/WOG groups (P<0.05). There was no significant difference between the WOG and PG/WOG groups (P>0.05). The WGG/WOG and PG/WOG groups were significantly faster than the WOG group.

**Conclusion.** The amount of debris extruded apically significantly diminished when conventional WGG was implemented before using WOG. The total preparation time significantly diminished when the WOG file was used in combination with reciprocating and rotary glide path preparation techniques.

## Introduction

A glide path, smoothly centered from the canal orifices to the physiological terminus, facilitates root canal preparation with Ni-Ti instruments by monitoring the tip of the first rotating instrument.^1-3^ During root canal preparation, complications such as taper lock, instrument fracture, and shaping aberrations, can be prevented by creating a glide path.^2-4,5^

Some studies have shown that the debris extrusion decreases, and the postoperative pain is less after using the pathfile.^6^

Although it is well established that all canal preparation techniques cause apical debris extrusion, the effect of the preparation technique on the amount of extruded debris continues to be studied.^7^ There are conflicting results regarding this issue; for example, Cakici et al^8^ and De-Deus et al^9^ demonstrated that the reciprocating system resulted in reduced apical extrusion of debris.However, Kuştarcı et al^10^ found no statistically significant difference between reciprocating and Twisted file systems in premolars.According to the meta-analysis of Pedrina et al,^11^ the reciprocating system gave rise to more successful results in molar teeth than continuous rotation systems.

The glide path files facilitate root canal preparation, especially in curved canals.^12,13^ Manual and mechanical techniques have been used for obtaining a glide path. When mechanical glide path preparation technique is used, there is an improvement in the preservation of the canal anatomy, a reduction in clinical chair time, a lower incidence of postoperative pain, and fewer canal aberrations than the manual glide path preparation technique.^5,6,14^

Many pathfinding Ni-Ti rotary instruments have been introduced in recent years. The Ni-Ti rotary glide path file ProGlider (Dentsply/Maillefer) is manufactured by using the memory Ni-Ti wire (M-Wire) technology. The ProGlider file has a square cross-section, a tip diameter of 0.16 mm, and a progressive taper from 2% to 8% over the cutting flute length.

The WaveOne Gold Glider (Dentsply Maillefer, Ballaigues, Switzerland) instrument introduces the reciprocating motion for glide path preparation. The lifespan of a NiTi instrument was shown to extend by reciprocating motion; it also increased fatigue resistance compared to continuous rotation.^15^ The use of the Gold wire technology, with two cutting edges and parallelogram cross-sectional design, improved this file. The WaveOne Gold Glider has an 0.15-mm diameter tip and variable 2–6% taper with maximum flute diameters of D1 = 0.170 mm, D8 = 0.413 mm, and D16 = 0.850 mm.^16^

This study purposed to evaluate the impact of reciprocating and rotary single glide path instruments on the amount of debris extrusion and preparation time during root canal shaping with WaveOne Gold in curved mesial root canals of mandibular molars.

## Methods

This study used lower first molar teeth extracted for periodontal causes. Radiographs were taken in both the buccolingual and mesiodistal aspects. Based on the inclusion criteria, mesial canal curvature angles were between 25° and 35° in the selected teeth, and the teeth had two separate mesial root canals and apical foramina. A periodontal curette was used to remove the dentin debris and remnants of soft tissue from the root surfaces. An endodontic access cavity was prepared for all the samples, followed by removing the distal root and the coronal part of each sample, using a high-speed diamond-coated bur under air**-**water spray at the furcation level. For standardization of the WL to 17 mm in each tooth, a high-speed bur with water cooling was used to flatten the tooth cusps. A pre-curved #10 K-type file (Dentsply Maillefer) was used to negotiate the selected curved canals. When the file tip was vaguely visible at the apical foramen, the distance between the file tip and the rubber stop was measured, and 0.5 mm was subtracted from this measurement to determine the WL. The teeth with an apical diameter greater than #10 were excluded from the study. Thirty-six fully formed mesial roots were selected and randomly splited into three groups (n=12).

### Debris collection

**Figure 1:**
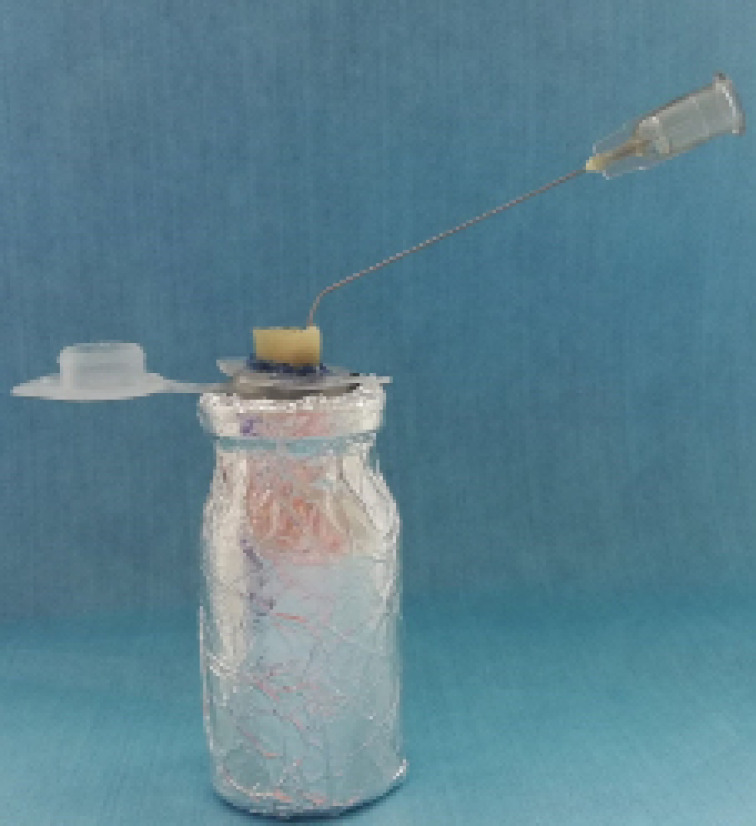
The experimental model system used to evaluate debris extrusion

A method of Myers and Montgomery^16^ was also modified for debris collection. An electronic weighing machine (Precisa XR 1255, Switzerland) was used to weigh the Eppendorf tubes with an accuracy of 10^-5^. To calculate the average weight of each tube, three consecutive measurements were made by the same operator. After the separation of stoppers, which were irrelevant to our study from each Eppendorf tubes, holes were made in the stoppers to immobilize the teeth during the experiment. The teeth were placed in the stoppers, up to the cementoenamel junction, and then cyanoacrylate (Pattex Super Glue; Istanbul, Turkey) was used to fix the teeth in order to prevent irrigant leakage. To equilibrate the air pressure inside and outside of the tube, a needle was placed through the stoppers (Figure 1). Under the rubber dam isolation with ligated thread, an aspirator was used to remove excessive irrigant from the tooth’s coronal portion.

### Root canal instrumentation

### WaveOne Gold Glider + WaveOne Gold (WGG+WOG)

WaveOne Gold Glider was used to create a glide path, and the root canals were prepared with WaveOne Gold Primary file at the WL. In this group, the instruments were used in a slow, reciprocating, in-and-out pecking motion with the WaveOne all program option of the endodontic motor (X-Smart Plus, Dentsply Sirona).

### ProGlider+WaveOne Gold (PG+WOG)

In this group, ProGlider file was used to create a glide path with a continuous rotation motion. The speed was 300 rpm, and the torque was 200 g/cm. WaveOne Gold Primary (#25, 08) was used for canal shaping following the glide path preparation.

### WaveOne Gold (WOG)

In this group, a glide path was not created , and the root canals were shaped by only WaveOne Gold Primary similar to that in other studies.

Totally, 10 mL of distilled water was used of each mesial canal in all teeth using an injector and a 29-gauge side-vented Navi Tip Irrigation needle (Ultradent, South Jordan, UT) for irrigation. After the irrigation of the root surface with 1 mL of distilled water inside the tube, the debris attached to the apical root surface was collected. An incubator was then used at 70ºC for five days to dry the contents in the Eppendorf tubes before weighing the dried debris. To achieve the average weights, three consecutive measurements were made for each eppendorf tube. To calculate the dry weight of the apically extruded debris, the weight of the empty tube was subtracted from the weight of the debris-containing tube.

### Preparation time

The total time consumed for the enlargement of this glide path and instrumentation of each root canal at WL was recorded using a digital chronometer by an assistant. The chronometer was started when the file was put into the canal and stopped when the instrument was retrieved. The time taken to change files was not recorded. Each file was used for the instrumentation of only three canals, and then the file was eliminated. The time during debris cleaning from the instrument flutes, irrigation, recapitulation, and re-irrigation was not involved in the total time. The preparation time of the glide path and shaping was recorded separately. Finally, the total preparation time was recorded for each tooth.

### Statistical analysis

Data obtained were statistically analyzed using SPSS (Statistical Package for Social Sciences 15.0 Chicago, IL, USA) software. Statistical analysis of the differences between the experimental groups was performed with One-Way variance analysis and the significance was evaluated at the level of p<0.05.

## Results

Table 1 presents the mean weight of the extruded debris and standard deviation of all the groups are presented. The WGG/WOG group extruded significantly less debris than the WO group (P<0.05). There was no significant difference between the WO and PG/WOG groups (P>0.05).

**Table 1:** Weight (mg) of apically extruded debris after preparation

**Groups**	**n**	**Mean Weight**	**SD**
**WOG**	12	0.0055025^a^	0.00201582
**WGG /WOG**	12	0.0032858^b^	0.00179985
**PG/WOG**	12	0.0053792^a^	0.00244684
**Total**	36	0.0047225	0.00228932

SD, standard deviation. Values with the same letters were not significantly different at P=0.05

Instrument fracture or root canal blockage did not cause any loss of specimens. Table 2 presents the mean preparation times and standard deviations of the preparation groups. The WGG/WOG and PG/WOG groups were faster than the WOG group at a statistically significant degree (P<0.05). No statistically significant difference was observed between the mean total preparation times of the WGG/WOG and PG/WOG groups (P<0.05).

**Table 2:** Descriptive Statistics for Total Preparation Time (Seconds) for the Different Groups

**Technique**	**Number**	**Mean**	**Standard Deviation**	**Minimum**	**Maximum**
**WOG**	12	35.9^a^	11.4	22.56	57.10
**WOG / WGG**	12	27.1^b^	4.4	20.52	32.84
**WOG/PG**	12	24.4^b^	3.4	19.55	29.84

SD, standard deviation. Values with the same letters were not significantly different at P=0.05.

## Discussion

This study interpreted the influence of novel reciprocating and rotating glide path files on the amount of apically extruded debris and preparation time during curved root canal shaping with WaveOne Gold System. In this study, extrusion of apical debris was present in all the experimental groups in which root canal preparations were compared. WGG/WOG group extruded less debris than WOG and PG/WOG groups. A study by Gunes et al^17^ on the influence of glide path preparation on the amount of apical debris extrusion showed similar results, as no significant difference was found between the WOG and PG/WOG groups.Topçuoglu et al^18^ showed that the creation of a glide path decreased apical extrusion of debris, and they used the path file system for creating a glide path.

In the present study, WaveOne Gold Glider and Proglider single file systems were used for glide path preparation, and the WGG file decreased the amount of apically extruded debris only. WaveOne Gold Glider is manufactured from Gold wire, which is resistant to failure due to cyclic fatigue compared to a file system made of M-wire.^19,20^ As a result, the WGG file was more flexible than the PG file. Highly flexible instruments might produce less apical transportation in curved canals. It is well known that apical transportation and irregular foramen enlargement can cause inadequate sealing efficiency with excessive debris extrusion.^21^ This might explain the reduced extrusion in the WGG group compared to the PG and WOG groups. Additionally, many properties of the files used, such as cross-sectional design, cutting efficacy, the diameter of tip and taper, motion kinematics, and the number of files in the system, influence the amount of apical debris extrusion.^22^ WaveOne Gold Glider also has the smallest tip diameter (0.15 mm).

A modified method of Myers and Montgomery^16^ was also used for collecting the debris without simulating the resistance of periapical tissue. But, in clinical settings, the periapical tissues, which are a natural barrier, play a key role in reducing the amount of extruded debris. The vital periapical tissue could not be imitated; thus, the results might have been affected in an in vivo model. Nevertheless the results should not be directly extended to clinical settings, the method can be used to collect apically extruded debris successfully. In the present study, distilled water was used as an irrigant to prevent sodium hypochlorite crystallization.

This study demonstrates that using a glide path with WaveOne Gold reduces the curved canals’ preparation time. Moreover, the total preparation time decreased. Berutti et al^12^ studied the preservation of canal curvature using a glide path, following the instrumentation with WaveOne primary reciprocating files. They showed that creating a glide path caused fewer pecking motions to reach the working length with WaveOne single files. The reason for this decrease in the total preparation time might be the use of these glide path files in WGG/WOG and PG/WOG. On the contrary, Coelho et al^23^ showed that creating a glide path increased the total preparation time, but they used #10, #15, and #20 hand K- type files to create the glide paths. Using stainless steel manual K- type files for creating a glide path was much slower than using rotary instrument groups.^24^ This observation explains the findings of a study by Coelho et al. Another study by Vorster et al^25^ reported that WGG performed the creating a glide path significantly faster than other glide path techniques. The author thought that this result could be attributed to the fact that WGG is a single file system.

However, there were no significant differences in the total final shaping times between the WaveOne Gold groups combined with different glide path files. The present study showed that the single file systems, WGG and PG, provided significantly faster preparation in terms of the final shaping time.

## Conclusion

Within the limitiations of our study , the creation of a glide path with the WaveOne Gold Glider and ProGlider before WaveOne Gold instrumentation decreased total preparation time. The other conclusion of the present study is that the amount of apically extruded debris decreased when using the WaveOne Gold Glider file combined with the WaveOne Gold Primary in curved canals.

## Conflict of Interests

The authors deny any conflict of interests.

## Authors’ Contributions

K. D. designed the study. D.K and K.S contributed to the material and method stage and writing stage. K.A and analysed the data. Both authors have read and approved the final manuscript. All the authors have read and agreed to the published version of the manuscript.

## Acknowledgments

There is no acknowledgements.

## Funding

Not applicable

## Ethics Approval

This article does not contain any studies with human participants or animals.
